# Prognostic Significance of Electrocardiography, Echocardiography, and Troponin in Patients Admitted With Non-ST Elevation Myocardial Infarction

**DOI:** 10.7759/cureus.37629

**Published:** 2023-04-16

**Authors:** Fawaz Bardooli, Dileep Kumar, Jehangir Hasan, Naeem Mengal, Khalid Iqbal Bhatti, Dayaram Makwana, Kelash Rai, Lalit Maheswari

**Affiliations:** 1 Interventional Cardiology, Mohammed Bin Khalifa Bin Salman Al Khalifa Specialist Cardiac Centre, Awali, BHR; 2 Cardiology, National Institute of Cardiovascular Diseases, Karachi, PAK; 3 Interventional Cardiology, National Institute of Cardiovascular Diseases, Karachi, PAK; 4 Cardiology, Mohammed Bin Khalifa Bin Salman Al Khalifa Specialist Cardiac Centre, Awali, BHR; 5 Internal Medicine, Wayne State University School of Medicine, Rochester Hills, USA; 6 Medicine, Global Medical Solutions Hospital Management LLC, Abu Dhabi, ARE

**Keywords:** : acute coronary syndrome, non-st segment elevation myocardial infarction (nstemi), cardiac troponin i, echo cardiogram, 12-lead ecg

## Abstract

Background: Non-ST segment elevation myocardial infarction (NSTEMI) is a clinical condition characterized by typical symptoms of myocardial ischemia along with electrocardiographic changes and a positive value of troponin. After presentation in the emergency department, these patients have their troponin I value and electrocardiography done. Echocardiography (echo) should also be performed on these patients. This study was conducted to determine the prognostic significance of ECG, echo, and troponin.

Methods: This observational study was conducted at a tertiary care cardiac hospital on 221 diagnosed patients of NSTEMI. Electrocardiography was performed to see any particular resting ECG findings and the peak values of cardiospecific troponin were analyzed for associations with major adverse events after a six-month period of follow-up. On echo, the left ventricular ejection fraction was divided into two categories: left ventricular ejection fraction (LVEF) <40% and LVEF >40%.

Results: The most frequent finding on presenting ECG was ST depression in anterior leads (V1-V6) in 27.6%. Median troponin I at presentation was 3.2 ng/dl and the median ejection fraction was 45%. The overall all-cause mortality rate at six months was observed to be 8.6%; re-infarction in 5%, re-hospitalization in 16.3%, and heart failure in 25.3% were observed. However, mortality was higher for patients with baseline ECG findings of A-fib, generalized ST-depression, poor R-wave progression, Wellens sign, and T-wave inversion in inferior; the mortality rate was also relatively higher among patients with poor LVEF (<30%).

Conclusion: ECG and echo were prognostically significant and with the combined incidence of adverse events. However, troponin lacks prognostic significance at six months.

## Introduction

Non-ST-segment elevation myocardial infarction (NSTEMI) is an acute coronary syndrome characterized by typical symptoms of myocardial ischemia and possible electrocardiographic changes along with troponin leak. In the emergency department, these patients have their troponin I value evaluated and electrocardiography is performed along with echocardiography (echo). These investigations are helpful in risk stratification to undergo coronary intervention or to manage conservatively.

Although all these modalities have a high impact on prognosis independently, there are no studies available that correlate the ECG, echo, and troponin together. Moreover, while admitting that ECG findings are valuable in the assessment of risk and predicting the prognosis in patients with NSTEMI [1,2], an ECG could have numerous findings including ST segment depression, T wave inversion, and also, most of the time, it could be normal or have nonspecific findings. Previous studies have shown that ST depression has adverse short-term and long-term cardiovascular outcomes [3,4]. Additionally, troponin is a biomarker that is preferable in the setting of acute coronary syndrome and differentiates from unstable angina, even though the management strategy is the same for both conditions, and there are multiple studies on the prognostic value of troponin in NSTEMI [5-10]. Subsequently, echo is the basic tool to further evaluate patients with NSTEMI to recognize segmental wall motion abnormalities [11, 12], and left ventricular ejection fraction (LVEF) along with other parameters. This would be helpful in deciding the further management plan.

Therefore, studies are needed that associate all the basic modalities in NSTEMI and show the joint significance of the outcome in these patients are needed for further risk stratification. This study, thus, was conducted to determine the prognostic significance between ECG, echo, and troponin. Such information would help physicians to categorize patients further with their management plan.

This article was previously posted to the medRxiv preprint server on April 27, 2020.

## Materials and methods

This observational study was conducted at a tertiary care cardiac hospital, the National Institute of Cardiovascular Diseases, in Karachi, Pakistan, from August 2019 to August 2020. The sample size of this study was calculated and approximated to be around 300 patients. All the patients diagnosed with NSTEMI were enrolled in this study. The patients who were not diagnosed with NSTEMI and who did not give consent were excluded from this study. ECG and troponin test were the basic tools along with echo.

This study was commenced after the approval of the Ethical Committee of the National Institute of Cardiovascular Diseases, Karachi, Pakistan (approval number: ERC-43/2019). Verbal consent was taken and documented from all the enrolled patients. Baseline features including age, gender, risk factors such as diabetes, hypertension, obesity, smoking, and family history of premature coronary artery disease were documented on the designed questionnaire along with the vitals of the patients.

ECG was performed to see any particular ischemic findings and troponin value was also categorized. On echo, LVEF was divided into two categories: LVEF < 40% and LVEF > 40%. These all findings were noted to correlate with each other and to determine the outcome. All the patients were followed up for six months and outcomes such as all-cause mortality, re-infarction, re-hospitalization, and heart failure were recorded.

Data analysis was performed with the help of IBM SPSS Statistics for Windows, Version 21.0 (Released 2012; IBM Corp., Armonk, New York, United States), descriptive summary such as mean ± SD, median (interquartile range (IQR)), and range (maximum - minimum) were calculated for continuous variables, and frequency and percentages were calculated for categorical variables.

## Results

A total of 221 patients with NSTEMI were included, out of which 76.9% (n=170) were male and the mean age was 57.63 ± 10.48 years with 20.4% (n=45) above 60 years of age. A majority (84.6%) of patients were in Killip class I at presentation and only 2.7% (n=6) patients were in Killip class III and no patients were in Killip class IV. The commonest co-morbid condition was hypertension (82.4%) followed by diabetes (41.6%) and smoking (26.2%). A sedentary lifestyle was reported by 24.9% (n=55) of the patients. The most frequent finding on presentation ECG was ST depression - anterior (27.6%) followed by T wave inversion - anterior (14%), 12.2% (n=27) of patients had non-specific changes, and 16.3% (n=36) patients had normal ECG. Median troponin I at presentation was 3.2 ng/dL (7.3-1.2 ng/dL) ranging from 0.06 ng/dL to 50 ng/dL. The median ejection fraction (EF) was 45% (55-35%) with a range of 15-70%. Demographic characteristics, baseline troponin I level, ECG changes, and echo findings are presented in Table 1.

**Table 1:** Demographic characteristics, baseline troponin I level, ECG changes, echocardiographic findings CAD: coronary artery disease; IQR: interquartile range; ESD: end-systolic dimension; EDD: end-diastolic diameter

Characteristics	Total
Total (N)	221
Gender	
Male	170 (76.9% )
Female	51 (23.1% )
Age (years)	57.63 ± 10.48
≤ 50 years	62 (28.1% )
51 to 65 years	114 (51.6%)
> 65 years	45 (20.4%)
Killip class at presentation	
I	187 (84.6% )
II	28 (12.7%)
III	6 (2.7%)
IV	0 (0%)
Risk factors	
Hypertension	182 (82.4% )
Diabetes	92 (41.6% )
Smoking	58 (26.2% )
Dyslipidemia	26 (11.8%)
Family history of CAD	23 (10.4%)
Obesity	17 (7.7%)
Sedentary lifestyle	55 (24.9%)
Troponin I (ng/dL)	
Mean ± SD	6.35 ± 8.59
Median (IQR)	3.2 [7.3 - 1.2]
Range (Max - Min)	50 - 0.06
Ejection fraciton (%)	
Mean ± SD	43.02 ± 12.13
Median (IQR)	45 [55 - 35]
Range (Max - Min)	70 - 15
ESD	
Mean ± SD	34.9 ± 7.47
Median (IQR)	33 [38 - 29]
Range (Max - Min)	66 - 22
EDD	
Mean ± SD	47.82 ± 6.11
Median (IQR)	48 [51 - 44]
Range (Max - Min)	69 - 33
Baseline ECG	
ST depression: anterior	61 (27.6%)
ST depression: iInferior	12 (5.4%)
ST depression: lateral	12 (5.4%)
ST depression: general	7 (3.2%)
T wave inversion: anterior	31 (14% )
T wave inversion: inferior	7 (3.2%)
T wave inversion: lateral	2 (0.9%)
Poor R wave	12 (5.4%)
Hyperacute T wave	5 (2.3% )
Wellens sign	6 (2.7%)
Atrial fibrillation	3 (1.4% )
Non-specific	27 (12.2%)
Normal	36 (16.3%)

The proportion of patients in the fourth quartile (>7.30 ng/dL) was more prominent for the patients with baseline ECG findings of poor R-wave, ST depression - generalized, and inferior ST depression, while, the lower quartile distribution of troponin I was more commonly observed with non-specific ECG changes, normal ECG, inferior T-wave inversion, lateral ST-depression, and in patients with anterior ST-depression.

Poor LVEF (%) can be seen with ECG findings of atrial fibrillation (Afib), poor R-wave, ST-depression - anterior, and ST-depression - inferior. LEVF was in the normal range for most of the patients with normal ECG and ST-depression - lateral. High troponin I level and low LVEF were found to be related to ECG changes of ST-depression - generalized, poor R-wave, and Afib. Low troponin I level and high LVEF were observed against ECG changes of ST-depression - lateral, T-wave inversion, Wellens sign, and normal ECG. Adverse cardiac event rate at six months for various ECG changes, troponin I quartiles, and EF categories are presented in Table 2.

**Table 2:** Adverse cardiac event rate at six months against ECG changes, troponin I, and ejection fraction

	Total (N)	Six-months outcome			
		All-cause mortality	Re-infarction	Re-hospitalization	Heart Failure
Overall	221	19 (8.6%)	11 (5%)	36 (16.3%)	56 (25.3%)
ECG					
ST depression: anterior	61	3 (4.9%)	3 (4.9%)	10 (16.4%)	17 (27.9%)
ST depression: inferior	12	0 (0%)	1 (8.3%)	1 (8.3%)	1 (8.3%)
ST depression: lateral	12	1 (8.3%)	0 (0%)	3 (25%)	4 (33.3%)
ST depression: general	7	2 (28.6%)	1 (14.3%)	3 (42.9%)	3 (42.9%)
T wave inversion: anterior	31	2 (28.6%)	3 (9.7%)	8 (25.8%)	7 (22.6%)
T wave inversion: inferior	7	1 (14.3%)	0 (0%)	1 (14.3%)	3 (42.9%)
T wave inversion: lateral	2	0 (0%)	0 (0%)	1 (50%)	1 (50%)
Poor R wave	12	2 (16.7%)	0 (0%)	0 (0%)	5 (41.7%)
Hyperacute T wave	5	0% (0)	0 (0%)	0 (0%)	0 (0%)
Wellens sign	6	1 (16.7% )	1 (16.7% )	1 (16.7%)	1 (16.7%)
Atrial fibrillation	3	1 (33.3%)	1 (33.3%)	2 (66.7%)	2 (66.7%)
Non-specific	27	3 (11.1% )	1 (3.7%)	4 (14.8%)	6 (22.2%)
Normal	36	3 (8.3%)	0 (0%)	2 (5.6%)	6 (16.7%)
Troponin I					
1st Quartile (≤1.20)	61	8 (14%)	6 (10.5%)	12 (21.1%)	18 (31.6%)
2nd Quartile (1.20 to 3.20)	12	2 (3.7%)	1 (1.9%)	6 (11.1%)	9 (16.7%)
3rd Quartile (3.20 to 7.30)	12	4 (7.3%)	4 (7.3%)	8 (14.5%)	14 (25.5%)
4th Quartile (>7.30)	7	5 (9.1%)	0 (0%)	10 (18.2%)	15 (27.3%)
Ejection fraction					
<30%	61	7 (35%)	3 (15%)	5 (25%)	12 (60%)
30 to 45%	12	11 (9.6%)	6 (5.2%)	26 (22.6%)	38 (33%)
> 45%	12	1 (1.2%)	2 (2.3%)	5 (5.8%)	6 (7%)

The overall all-cause mortality rate at six months was observed to be 8.6%, re-infarction rate 5%, re-hospitalization 16.3%, and heart failure rate 25.3% was observed. The all-cause mortality rate was relatively higher for patients with baseline ECG findings of Afib, ST-depression - generalized, poor R-wave, Wellens sign, and T-wave inversion in the inferior leads. The mortality rate was also relatively higher among patients with poor (<30%) LVEF. The relationship between mortality rate and troponin I level is non-conclusive.

Re-infarction rate was also relatively higher for the patients with ECG findings of Afib, Wellens sign, and generalized ST depression. Re-infarction rates surprisingly tend to remain higher for the lower quartile of troponin I. Poor LVEF was also observed to be associated with a higher re-infarction rate. The heart failure rate was observed to be related to poor LVEF and baseline ECG findings of Afib, ST-depression - generalized, poor R-wave, T-wave inversion inferior. However, the relationship between troponin I and heart failure rate remains inconclusive.

## Discussion

This study shows a recent evaluation of patients with NSTEMI in the contemporary era according to the presentation of ECG findings along with troponin level and echo in a large tertiary care center in Pakistan. The most significant finding on ECG in the present study was ST depression and T wave inversions in anterior leads that collectively accounted for 41.6% (ST depression anterior 27.6%, anterior T wave inversion 14 %) of total patients diagnosed with NSTEMI. This was in contrast with one of the large studies by Patel et al. that showed no ischemic changes (60.2%) as the most frequent finding [13]. However, our results were in line with a sub-analysis from the Global Use of Strategies to Open Occluded Arteries in Acute Coronary Syndromes (GUSTO-IIb) trial, where out of 12,142 patients, 35.1% had ST-depression, versus 22.4% with T-wave inversion [1]. A further greater frequency of ST depression was noted in the Invasive versus Conservative Treatment in Unstable Coronary Syndromes (ICTUS) trial, in which approximately 50% of the population had ST-depression at first presentation [14].

Moreover, ECG findings of generalized ST segment depression and poor R wave progression were better correlated with the high troponin value and low EF and resulted in a poor prognosis at six months. All-cause mortality was higher in afib with NSTEMI (33.3%), followed by generalized ST segment depression (28.6%), Wellens sign (16.7%), poor R wave progression (16.7%). Heart failure, re-infarction, and re-hospitalization were high in afib, generalized ST depression, and poor progression of R wave followed by other findings.

In this study, the mean range of troponin was 6.35+-8.59 and the mean EF was 43.02 ± 12.13%. Troponin level is a good predictor of mortality but the results showed in this study are inconclusive. However, a study by Yariv et al. showed Troponin T values at 30 days were 5.8 (95%CI, 1.4-10.2) for death, 5.2 (95% CI, 0.2-10.3) for recurrent ischemic events, and 6.9 (95%CI, 1.4-12.4) for heart failure [15]. This study revealed the significance of a low ejection fraction of < 30% resulting in overall mortality of 35% in comparison to the EF of 30-45 and > 45%, which had 9.6% and 1.2%, respectively.

ECG, echo, and troponin altogether categorize the patients according to the risk of adverse events to the sixth month after NSTEMI.

This study has a few limitations. As this was a single-center study and had a small sample size, it cannot be generalized. Multi-center large-volume studies are required to generalize to the whole population.

## Conclusions

ECG, echo, and troponin have better prognostic significance at six months. However, troponin is considered of high importance in predicting outcomes and is an independent factor. Furthermore, ECG and echo are correlated well with each other in patients with NSTEMI. These three variables are usually performed in every patient and are important to stratify the patients for further management.
